# Membrane Lipid Remodelling of *Meconopsis racemosa* after Its Introduction into Lowlands from an Alpine Environment

**DOI:** 10.1371/journal.pone.0106614

**Published:** 2014-09-03

**Authors:** Guowei Zheng, Bo Tian, Weiqi Li

**Affiliations:** 1 Key Laboratory for Plant Diversity and Biogeography of East Asia, Kunming Institute of Botany, Chinese Academy of Sciences, Kunming, Yunnan, People's Republic of China; 2 Key Laboratory of Tropical Plant Resource and Sustainable Use, Xishuangbanna Tropical Botanical Garden, Chinese Academy of Sciences, Kunming, People's Republic of China; 3 Plant Germplasm and Genomics Center, Germplasm Bank of Wild Species, Kunming Institute of Botany, Chinese Academy of Sciences, Kunming, Yunnan, People's Republic of China; National Taiwan University, Taiwan

## Abstract

Membrane lipids, which determine the integrity and fluidity of membranes, are sensitive to environmental changes. The influence of stresses, such as cold and phosphorus deficiency, on lipid metabolism is well established. However, little is known about how plant lipid profiles change in response to environmental changes during introduction, especially when plants are transferred from extreme conditions to moderate ones. Using a lipidomics approach, we profiled the changes in glycerolipid molecules upon the introduction of the alpine ornamental species *Meconopsis racemosa* from the alpine region of Northwest Yunnan to the lowlands of Kunming, China. We found that the ratios of digalactosyldiacylglycerol/monogalactosyldiacylglycerol (DGDG/MGDG) and phosphatidylcholine/phosphatidylethanolamine (PC/PE) remained unchanged. Introduction of *M. racemosa* from an alpine environment to a lowland environment results in two major effects. The first is a decline in the level of plastidic lipids, especially galactolipids. The second, which concerns a decrease of the double-bond index (DBI) and could make the membrane more gel-like, is a response to high temperatures. Changes in the lipidome after *M. racemosa* was introduced to a lowland environment were the reverse of those that occur when plants are exposed to phosphorus deficiency or cold stress.

## Introduction

Plant introduction and acclimatization, the products of which now account in large part for many of our foods and ornamental species, played a critical role in the emergence of civilisation [1]. During the process of introduction, plants are transferred from their native environments to artificial ones where resources are usually plentiful and the plants can avoid stresses, such as freezing, drought, nutrient deprivation, and infection with pathogens [1], [2]. The major obstacle to successful introduction is whether plants can adapt to dramatic changes in their environment. For example, the introduction of alpine plants to a lowland environment for the purpose of preservation and sustainable use is very difficult, because few plants can overcome considerable changes in temperature, irradiation, water conditions, and even nutrition [3]–[5]. Most studies of plant adaptation to environmental changes have focused on the adaption to stresses in which environments shift from optimum to adeverse conditions [6], [7]. The mechanisms that plants use to adapt to moderate environments after their transfer from extreme environments are not fully understood. Given that the responses of an organism to two opposite stimuli are often not simply the direct inverses of each other, understanding how plants adapt to the transfer from alpine to lowland conditions is an issue of biological significance and commercial importance.

Plants can adapt to environmental changes by adjustments at the morphological, physiological, biochemical, and molecular levels [6]–[8]. Membranes are integral to the structure and function of all cells; maintenance of the integrity and fluidity of membranes is of fundamental importance if plants are to survive environmental changes [9]–[11]. Glycerolipids are the major constituents of membranes. Lipid remodeling, through adjustment of the composition, unsaturation (represented by the double-bond index, DBI) and the acyl chain lengths (ACL) of their constituent fatty acids, is one of the most important ways that plants use to maintain the function of membranes upon exposure to fluctuating environmental conditions. Plants tend to synthesise additional galactolipids to replace phospholipids under conditions of phosphate deficiency [12]–[14], but to increase the proportion of phospholipids in response to low temperatures [15], [16]. Membrane lipids, such as DGDG and phosphatidylcholine (PC), have relatively large polar head groups that tend to form membranes with the lamellar phase (Lα), which can enhance the stability of the membrane under various stresses. In contrast, monogalactosyldiacylglycerol (MGDG) and phosphatidylethanolamine (PE)—which have relatively small head groups—show a higher propensity for transition to the non-bilayer H_II_-type structures. The increased ratio of DGDG/MGDG and PC/PE could enhance the stability of the membrane under temperature and dehydration stresses [17]–[20].

Changes of the DBI and ACL of membrane glycerolipids that enable the fluidity of membranes to be adjusted are other important responses of membranes to stress, especially that caused by temperature extremes. Low temperature results in a 31% increase in the degree of unsaturation of fatty acids [21]. In contrast, the degree of unsaturation of fatty acids in plants decreases following exposure to high temperatures. For example, the DBI of *Arabidopsis* plants grown at 36°C was 39% lower than that of plants grown at 17°C [22]. Alternatively, whereas longer-chain fatty acids can make the membrane environment more gel-like, shorter chains help to maintain the fluid state of membranes [23]. As such, in bacteria, it is common to see a decrease in the average length of fatty acyl chains as the growth temperature decreases [24].

In alpine-scree ecosystems of the Baima Snow Mountain in Northwest China, the daytime temperature exceeds 35°C, whereas the temperature at night can drop below freezing [5]; in addition, the level of available phosphorus is very low (1.3 ppm) [25]. *Meconopsis racemosa*, a member of the Papaveraceae, is a native of the alpine scree of the Baima Snow Mountain. It is a well-known horticultural plant that bears beautiful flowers. However, the genetic resources of this plant are threatened by habitat destruction. Its introduction into other areas outside of its native habitat is thus one of the most important approaches to protect this ornamental plant. A factor that might limit the introduction of *M. racemosa* into other environments is its poor photosynthetic performance at high temperatures (>30°C) [26], [27]. The biochemical responses of *M. racemosa* upon its transfer outside of its native habitat remain unknown, especially in terms of membrane lipid profiles.

The process of introducing *M. racemosa* from an extreme alpine environment to a moderate lowland environment involves exposure to substantially different environmental conditions, including considerable changes in temperature and the availability of soil nutrients. This process is different from the study of stress response where plants are transferred from moderate to extreme environments. It remains to be established whether the patterns of change of lipid profiles following transfer from extreme to moderate conditions are the reverse of those that occur upon exposure to the stress caused by transfer from moderate to extreme conditions.

Plant lipidomics based on ESI-MS/MS is a useful method to study the responses of hundreds of lipid molecular species to various environmental stresses [5], [20]. The purposes of the current study were to use lipidomics to characterise the changes in *M. racemosa* lipids that occur during its introduction from alpine scree to the lowlands of Kunming, and to compare the lipid remodeling observed when plants are transferred from extreme to moderate environments with those that occur in response to specific environmental stresses.

## Materials and Methods

### Study site and plant materials

The study site comprised alpine scree at an altitude of 4,560 m on the Baima Snow Mountain in the Hengduan Mountains, a mountain range in Southwest China in the Southeast Qinghai-Tibet Plateau. The collection of samples were permitted by The Forestry Department of Yunnan Province. In this ecosystem, the solar radiation is very strong, and the temperature changes dramatically from about 32°C in the daytime to 3°C at night [5]. In the lowland of Kunming where *M. racemosa* introduced, the altitude is 1,900 m, and the average temperature in the greenhouse during July is approximately 25/15°C (day/night).

*M. racemosa* Maxim (Papaveraceaehttp://zh.wikipedia.org/w/index.php?title = Arctomecon&action = edit&redlink = 1) is an herb with a height of 20–50 cm, which grows on stony slopes at an altitude of 3,000–4,600 m. It has sharp spines on its leaves and stems, and the stem is branched with many blue or purple flowers. Its seeds are oblong (1–2 mm long on their longest axis) and ripen around September (http://www.efloras.org/florataxon.aspx?flora_id=2&taxon_id=242331771). It is not an endangered or protected species. Field sampling was performed at the alpine study site (28° 23.265′ N, 099° 01.260′ E, 4502 m alt.) on a randomly chosen day in July 2005. The leaves were transferred immediately into 3 mL of isopropanol with 0.01% butylated hydroxytoluene in a boiling water bath for at least 15 min at an altitude of 4,502 m. In the laboratory experiment, seeds of *M. racemosa* Maxim were surface-sterilised; then, after five days incubation at 4°C, the seeds were sown in soil, germinated, and grown in a greenhouse. After three months of growth, lipids were extracted from leaves immediately after their excision.

### Lipid extraction, ESI-MS/MS analysis and data processing

Lipid extraction, ESI-MS/MS analysis, and quantification were performed as described previously, with minor modifications [5], [20]. To inhibit lipolytic activity, harvested leaves were transferred immediately into 3 mL of isopropanol with 0.01% butylated hydroxytoluene in a boiling water bath (field experiment) or a 75°C water bath (laboratory experiment). The leaves were extracted three times with a chloroform:methanol (2∶1) mixture, with 12 h of agitation each time. The remaining plant tissue was dried overnight at 105°C and weighed to determine the dry weights of the plants. Lipid samples were analysed using a triple quadrupole MS/MS equipped for ESI. Data processing was performed as described previously [20]. The lipids in each plant were quantified by comparison with two internal standards for the class of lipid studied. Five replicates of each treatment for each plant were analysed. The Q-test was performed on the total amount of lipid in each class, and data from discordant samples were removed [20]. The data were subjected to one-way analysis of variance (ANOVA) with SPSS 13.0. Statistical significance was tested by Fisher's least significant difference (LSD) method. DBI was calculated using the following formula: DBI  =  (∑[*N* × mol% lipid])/100, where *N* is the number of double bonds in each lipid molecule [5]. ACL was calculated using the following formula: ACL  =  (∑[*n* × mol% lipid])/100, where n is the number of acyl carbons in each lipid molecule.

## Results

### Major environmental changes associated with the introduction of *M. racemosa* from alpine scree to a lowland environment

**Figure 1:**
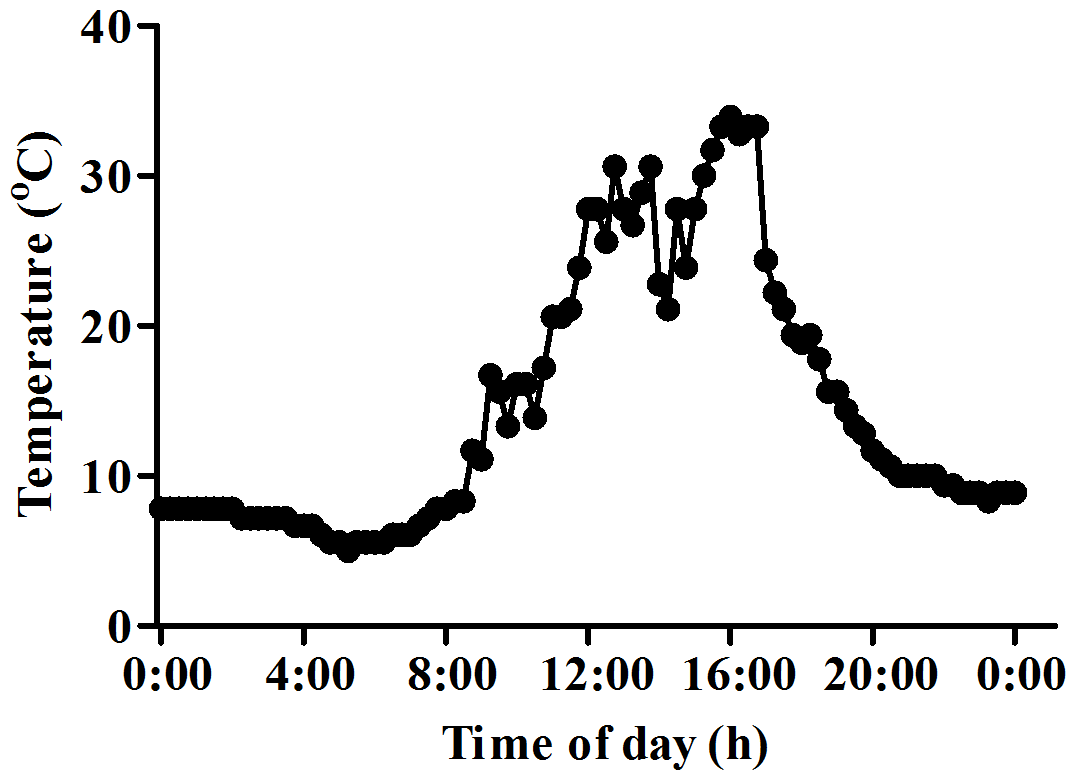
Variation in air temperature with time of day in alpine screes of the Hengduan Mountains in July. The temperature was recorded every 30 min on a clear day in July.

The microclimate at the alpine study site where *M. racemosa* grows was investigated during July. This mainly involved examining the variations in temperature (Fig. 1), solar radiation, and UV radiation within a 24-h period on a clear day. *M. racemosa* growing on alpine scree experienced a dramatic change in temperature over the course of a day, from 33°C in the daytime to 5°C at night. On the day of sampling, the average air temperature was 14.6°C (Fig. 1). Compared with alpine scree, the temperature in the greenhouse in Kunming was about 25/15°C (day/night), with an average of 20°C. The soil in the alpine scree was previously shown to be deficient in nitrogen, potassium and phosphorus, with phosphorus available at a level of only 1.3 ppm, which is very low [25]; in contrast, in the lowlands in Kunming where *M. racemosa* was introduced, the plants were watered with 1/4 Hoagland solution once every month, and the level of phosphorus was sufficient to support normal growth of *M. racemosa*. Considering the differences in microclimate between the alpine scree of Baima Snow Mountain and the lowlands where the greenhouse is situated, this introduction of *M. racemosa* could be described as a process in which plants were introduced from an extreme environment to a moderate one. As one major morphological change associated with this introduction, it was found that leaves of *M. racemosa* grown in the greenhouse were larger than those from the alpine scree (data not shown).

### Significant changes in the levels of membrane glycerolipid molecules occurred in *M. racemosa* after its introduction into the Kunming lowlands

**Figure 2:**
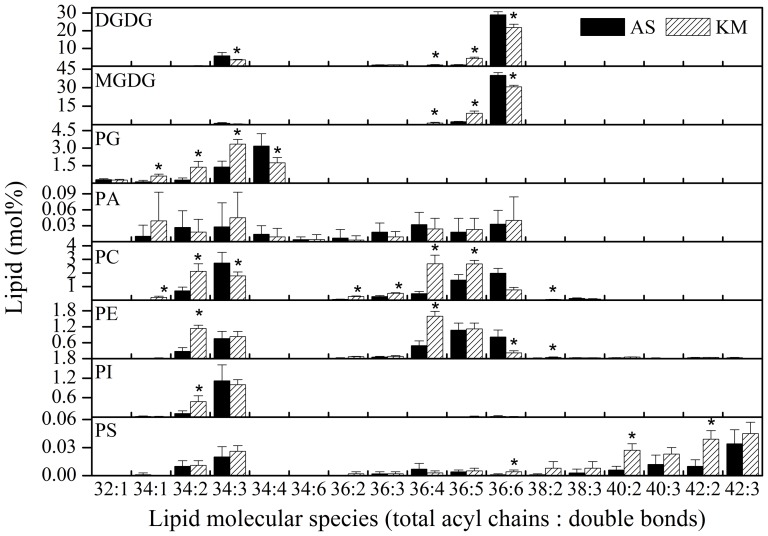
Changes in the molecular species of membrane lipids in *Meconopsis racemosa* grown in alpine scree (AS) and Kunming (KM). An asterisk indicates that the value of KM is significantly different from that of AS (*P*<0.05). Values are means ± standard deviation (*n* = 4 or 5).

**Figure 3:**
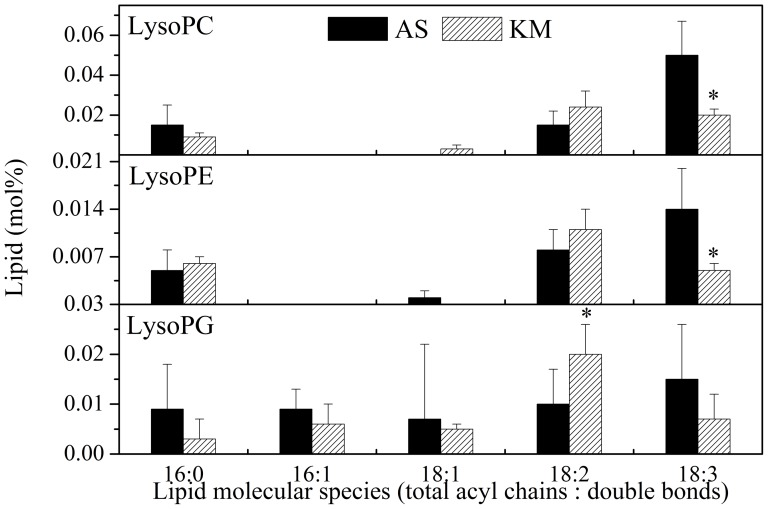
Changes in the molecular species of lysoPLs in *Meconopsis racemosa* grown in alpine scree (AS) and Kunming (KM). An asterisk indicates that the value of KM is different from that of AS (*P*<0.05). Values are means ± standard deviation (*n* = 4 or 5).

An ESI-MS/MS-based lipidomics approach [5], [20] that can identify and quantify 11 classes of 130 molecular lipid species was used to examine the patterns of lipid changes in *M. racemosa* during its introduction into a lowland environment. The 11 classes comprise two classes of galactolipids, six classes of phospholipids (Fig.2), and three classes of lysophospholipids (lysoPLs) (Fig.3). The observation that *M. racemosa* contains only 36∶6 MGDG molecules (Fig.1) indicates that it is an 18∶3 plant that harbours only the eukaryotic lipid synthesis pathway [28].

Detailed analysis of the lipid profiles indicated that the levels of many lipid molecules, but not PA, changed significantly after *M. racemosa* was introduced from an alpine habitat to the Kunming lowlands (Fig.1). Most lipid classes tended to include lipid molecules with fewer double bonds after *M. racemosa* was introduced to a lower altitude; for example, the level of 36∶5 DGDG lipid molecules increased almost six-fold (from 0.76 to 4.43 mol%), whereas that of 36∶6 DGDG decreased by 25% (from 29.04 to 21.72 mol%). In addition, the level of 36∶5 MGDG increased almost four-fold (from 2.42 to 9.22 mol%), whereas that of 36∶6 MGDG decreased by 24% (from 40.10 to 30.63 mol%). Levels of 36∶4 PC increased more than five-fold (from 0.48 to 2.67 mol%), those of 36∶6 PC decreased by 62% (from 1.98 to 0.75), those of 36∶4 PE increased more than three-fold (from 0.49 to 1.59 mol%), and those of 36∶6 PE decreased by 73% (from 0.81 to 0.22 mol%). The relative increase of PS was 75% (Table 1); in general, the content of most PS molecules increased dramatically, especially for lipids with long acyl chains, such as 40∶2 and 42∶2 PS (Fig.1).

**Table 1:** Leaf membrane lipid composition in each head group class and lipid ratios of *M. racemosa* grown in alpine scree (AS) and in Kunming (KM).

Lipid class	mol%		Relative change (%)(%)
	AS	KM	
DGDG	36.99±2.93	31.86±1.56^*^	−13.87
MGDG	44.16±2.49	42.06±1.35	−4.76
PG	5.34±1.55	7.44±1.20^*^	39.33
PA	0.19±0.10	0.22±0.16	15.79
PC	8.06±1.81	11.24±0.92^*^	39.45
PE	3.71±0.96	5.32±0.60^*^	43.40
PI	1.28±0.56	1.54±0.23	20.31
PS	0.12±0.05	0.21±0.04^*^	75.00
LysoPC	0.08±0.03	0.06±0.01	−25.00
LysoPE	0.03±0.01	0.02±0.00	−33.33
LysoPG	0.05±0.02	0.04±0.01	−20.00
	Lipid ratio		
PC/PE	2.19±0.13	2.13±0.28	−2.74
DGDG/MGDG	0.84±0.07	0.76±0.05	−9.52
Galactolipids/Phospholipids	4.59±1.51	2.84±0.20^*^	−38.13

The relative change in lipids after introduction of *M. racemosa* to KM is the percentage value for the difference between the values of AS and KM, divided by the value of AS. An asterisk indicates that the value of KM is different from that of AS (*P*<0.05). Values are means ± standard deviation (*n* = 4 or 5).

The lysoPLs—which include lysoPC, lysoPE and lysoPG—are derived from the hydrolysis of phospholipids at the sn-1 or sn-2 position of the glycerol backbone. Upon exposure to low temperature stresses, lysoPLs usually increase by 5- to 20-fold within hours or even minutes [15], [20]. Compared with *M. racemosa* in alpine scree, the same species cultured in the Kunming lowlands did not show significant changes in the levels of lysoPL species, except for some individual molecular species, such as 18∶3 lysoPC, 18∶3 lysoPE, and 18∶2 lysoPG. These results might indicate that *M. racemosa* uses lipid remodelling to acclimate to the environmental changes associated with introduction from an extreme to a moderate environment.

### Changes in the composition of lipid classes after introduced from an alpine environment to a lowland area

After the introduction of alpine *M. racemosa* to Kunming, the levels of two galactolipids and three lysoPLs decreased, whereas the contents of six phospholipids increased. The content of DGDG decreased significantly from 36.99% to 31.86%, and the content of MGDG decreased from 44.16% to 42.06% (Table 1). The contents of PC and PE increased 3.18% and 1.61%, respectively. The content of PG also increased, from 5.35% to 7.44% (Table 1). The ratio of galactolipids/phospholipids decreased from 4.59 to 2.84; this constitutes a decrease of 38% (Table 1). These results indicate that *M. racemosa* tends to synthesise more phospholipids after its introduction to a lowland environment. Investigation of the ratios of PC/PE and DGDG/MGDG revealed that the changes of these ratios were not statistically significant (Table 1). This result might suggest that membrane integrity was not disrupted during the introduction of *M. racemosa*, despite the major environmental differences between the two habitats.

### Different performance of plastidic and extraplastidic lipids upon introduction into a lowland area

DGDG, MGDG and some PG species are plastidc lipids which mainly located in photosynthetic membrane, whereas PC, PE, PA, PI, PS and other PG species which mainly located in plasma membrane are called extraplastdic lipids. Given that DGDG and MGDG are the most abundant plastidic lipids in the leaves of *M. racemosa*, a decrease in their levels represents a decrease in the total level of plastidic lipids. To further compare lipid changes between plastidic and extraplastidic membranes, the changes in the levels of molecular species of PG were analysed. Whereas 34∶4 PG (which harbours a 16∶1 acyl chain) is part of the plastidic membrane, both 34∶1 and 34∶2 PG are extraplastidic lipids. Of the two molecules that correspond to 34∶3 PG, one contains a 16∶1 acyl chain and is part of the plastidic membrane, whereas the other is extraplastidic [20], [29]. Among the five species of PG molecules that were tested, only the level of 34∶4 PG decreased considerably, namely, by 45.28%. In contrast, the levels of two plastidic lipids, 34∶1 and 34∶2, increased more than four-fold (Table 2). The content of 34∶3 PG increased almost three-fold, which was a smaller increase than those of the two plastidic lipids. These results indicate that the levels of extraplastidic lipids increased and plastidic lipids decreased after the introduction of the plant from an alpine environment to a lowland habitat.

**Table 2:** Levels of PG molecular species in leaves of *M. racemosa* plants.

PG species	mol%		Relative change (%)
	AS	KM	
32∶1	0.30±0.08	0.26±0.07	−13.33
34∶1	0.13±0.12	0.61±0.16^*^	369.23
34∶2	0.27±0.18	1.37±0.49^*^	407.41
34∶3	1.38±0.51	3.34±0.40^*^	142.02
34∶4	3.18±1.05	1.74±0.45^*^	−45.28

The relative change in PG species after introduction of *M. racemosa* to KM is the percentage value for the difference between the values of AS and KM, divided by the value of AS. An asterisk indicates that the value of KM is significantly different from that of AS (*P*<0.05). Values are means ± standard deviation (*n* = 4 or 5).

### Changes in the total degree of unsaturation and acyl chain length after the introduction of *M. racemosa* to the Kunming lowlands area

Changes in the degree of saturation and the lengths of fatty acid chains are very important for cells to modulate the fluidity of their membranes [23]. In this study, DBI was used to indicate the degree of unsaturation of membrane glycerolipids and ACL to indicate the lengths of fatty acid chains [5]. For all glycerolipids, the DBI of *M. racemosa* cultured in Kunming was less than that of plants grown in alpine scree, except for DGDG and PA, which showed no significant differences after introduction to the lowland environment. For plants grown in the Kunming lowlands, MGDG, which is one of the predominant lipid constituents of membranes (Table 1), had a DBI of 0.19, which is less than that of plants grown in the alpine scree (Table 3). The DBI of PE decreased by 0.72 after introduction of the plant, with values of 4.31 and 3.59 for the alpine scree and Kunming, respectively. Furthermore, the DBI of total lipids in the alpine scree was 5.30, whereas that for Kunming was 4.95 (Table 3). Except for PS, the ACL of most glycerolipids was not affected by the introduction of *M. racemosa* from an alpine environment to a lowland habitat. The ACL of PS increased from 38.52 to 39.45 after introduction to the lowlands (Table 3). These results suggest that *M. racemosa* changes the fluidity of its membranes to adapt to the environmental changes associated with its introduction into the Kunming lowlands, and that it does this primarily through adjusting of the level of unsaturation of its membrane lipids.

**Table 3:** DBI and acyl chain length of membrane lipids of *M. racemosa* after its introduction from an alpine habitat to a lowland habitat.

Lipid class	Growth Site	DBI	Acyl chain length
DGDG	AS	5.41±0.13	35.68±0.08
	KM	5.34±0.05	35.76±0.02
MGDG	AS	5.85±0.03	35.95±0.02
	KM	5.66±0.07^*^	35.97±0.01
PG	AS	3.31±0.15	33.86±0.06
	KM	2.77±0.09^*^	33.90±0.02
PA	AS	3.71±0.96	35.12±0.42
	KM	3.82±0.60	35.13±0.40
PC	AS	4.10±0.06	35.24±0.05
	KM	3.67±0.08^*^	35.32±0.04^*^
PE	AS	4.31±0.21	35.67±0.09
	KM	3.59±0.06^*^	35.42±0.05^*^
PI	AS	2.93±0.07	34.04±0.03
	KM	2.72±0.14^*^	34.07±0.03
PS	AS	2.89±0.08	38.52±0.72
	KM	2.69±0.04^*^	39.45±0.38^*^
Total	AS	5.30±0.03	35.62±0.02
	KM	4.95±0.06^*^	35.60±0.03

DBI  =  (∑[*N* × mol% lipid])/100, where *N* is the number of double bonds in each lipid molecule. ACL was calculated using the following formula: ACL  =  (∑[*n* × mol% lipid])/100, where n is the number of acyl carbons in each lipid molecule. An asterisk indicates that the value of KM is different from that of AS (*P*< 0.05). Values are means ± standard deviation (*n* = 5).

## Discussion

Given that membrane glycerolipids are a major component of membranes, the roles of their remodelling under stressful conditions, such as extreme temperatures and phosphorus starvation, have been one focus of research [5], [17], [30]–[33]. However, the consequences of introducing alpine plants to lower altitudes differ from those associated with stress in that the plants experience environmental changes from extreme to moderate conditions. In this study, we profiled the molecular species of lipid in alpine *M. racemosa* during its introduction into a lowland area, and calculated the DBI and ACL of plant membrane glycerolipids. Our results indicated that the ratio of bilayer-stabilising lipids to nonbilayer lipids and ACL of glycerolipids were maintained. There were two major changes with respect to lipid remodelling after *M. racemosa* was introduced into the lowland habitat. The first effect was a significant increase in the levels of phospholipids. The second was a significant decrease in the degree of unsaturation of most glycerolipids. These results might suggest that *M. racemosa* might adjust the composition of different lipids classes and the degree of unsaturation of glycerolipids to adapt to environmental changes after its introduction from an alpine environment to a lowland habitat.

Plastidic lipids, which contain unusually high content of trienoic fatty acids, are the main component of photosystems I and II [34]. Galactolipids, which are the major component of plastidic lipids, harbour more trienoic fatty acids than other membrane phospholipids; for example, the lipid molecules of 36∶6 DGDG that were tested here harbour two 18∶3 fatty acids [20]. The content of trienoic fatty acids in plants is closely related to their photosynthetic performance at temperature extremes. Plants indigenous to cold areas tend to have a higher content of trienoic fatty acids, and the photosynthesis of plants that have relatively high levels of chloroplast trienoic fatty acids is more sensitive to heat treatment than those with low levels of chloroplast trienoic fatty acids [35]. Although *M. racemosa* maintained a high level of trienoic fatty acids in the galactolipids fraction when grown in the alpine environment, the level of trienoic fatty acids declined after the species was introduced into the lowland environment (Fig. 2). This might contribute to the poor photosynthetic performance of *M. racemosa* at 30°C compared with that at 20°C [26]. The lower level of trienoic fatty acid after introduction to lowland conditions might be an adaptation to the higher temperature in the lowland habitat than in the alpine habitat.

Both DGDG and PC have relatively large head groups, and tend to form bilayer lipid phase. By contrast, MGDG and PE have small head groups, involved in the formation of a nonbilayer lipid phase [17], [20]. Adjusting the molar ratio of these lipids is one of the most important ways that plants use to respond to stresses. The molar ratio of PC/PE decreased in plants subjected to cold and dehydration stresses [19], [20], and an increase in the molar ratio of DGDG to MGDG enhances the stability of thylakoid membranes at high temperatures [18]. Notwithstanding the considerable difference in the ambient temperature experienced by *M. racemosa* after its introduction from an alpine to a lowland environment, there were no statistically significant differences in either PC/PE or DGDG/MGDG ratios between *M. racemosa* grown in an alpine habitat and those grown in a lowland environment (Table 1). The difference between these observations and those for plants subjected to environmental stress might suggest that membranes of *M. racemosa* might retain their integrity after introduction into a new environment to ensure adaption to major environmental changes.

The ratio of galactolipids to phospholipids was lower when *M. racemosa* was grown in the lowland habitat than when it was grown in the alpine environment (Table 1), and the content of DGDG decreased significantly after introduction to the lowland habitat (Table 1). Plants replace phospholipids with nonphosphorous galactolipids and transport them to extraplastidic membranes under phosphate starvation, which is very important for various physiological processes [13], [14], [31]. Whereas the level of phosphorus availability in Baima Snow Mountain (1.3 ppm) is very low [25], the level of phosphorus in the lowland area into which *M. racemosa* was introduced is sufficient to support its growth. The conversion of existing phospholipids to DGDG (recycling pathway) and the synthesis of new DGDG via the Kennedy (*de novo*) pathway are two routes that enable the remodelling of membrane lipids under conditions of phosphate starvation [32]. Alpine-grown *M. racemosa* might synthesise more DGDG to replace phospholipids in order to ensure the efficient use of the limited amount of available phosphorus. However, during its long-term evolution, this plant might have adapted to the limited availability of phosphorus in alpine areas by deriving DGDG exclusively via the Kennedy pathway. After it was introduced from an extreme to a moderate environment, where the level of phosphorus is sufficient, *M. racemosa* selectively synthesised more extraplastidic lipids and less plastidic lipids. The way in which lipids were remodelled after *M. racemosa* was moved from phosphorus-deficient to phosphorus-sufficient conditions was the reverse of the process observed in plants exposed to phosphorus starvation.

The DBI of membrane lipids is sensitive to temperature changes [23]; it tends to increase in order to maintain the fluid state of membrane under low-temperature treatment, and to decrease to make membranes more gel-like in order to cope with heat treatment [21], [22], [36]. The DBI of membrane lipids of *M. racemosa* grown under alpine conditions decreased after its introduction to a lowland environment (Table 3). The average temperature at the alpine scree in July was previously reported to be 7.4°C [25], and it was 20°C under the greenhouse conditions used in our study. It was thus proposed that the high degree of membrane saturation would help *M. racemosa* to cope with the low temperature of the alpine scree, and the increased DBI in lowland-grown *M. racemosa* was a response to the relatively high temperature in that habitat.

The present study revealed substantial changes in the lipid profiles of *M. racemosa* as they adapted to a lowland environment. The decreases in the level of galactolipids and DBI were mainly responses to phosphorus-sufficient and the relatively high temperature conditions, which are the opposite of the responses of lipids to phosphorus starvation and cold stresses. This suggests that alpine plants have the genetic potential to adapt to lowland environments through adjusting their membrane lipid composition. However, many issues on this topic remain unresolved. For example, further analysis is needed to elucidate the relationship between the low level of plastidic lipids in lowland plants and their reduced capacity for photosynthesis compared with plants grown at higher altitudes.
